# Conversations About Stillbirth Risk in Routine Antenatal Care: A Qualitative Study Post‐Implementation of the Safer Baby Bundle

**DOI:** 10.1111/1471-0528.18330

**Published:** 2025-08-13

**Authors:** Christine Andrews, Ashley Pade, Fran Boyle, Dan Richard Fernandez, Laura Singline, Ann Lancaster, David Alan Ellwood, Adrienne Gordon, Vicki J. Flenady

**Affiliations:** ^1^ Centre of Research Excellence in Stillbirth, Mater Research Institute The University of Queensland Brisbane Queensland Australia; ^2^ Institute for Social Science Research The University of Queensland Brisbane Queensland Australia; ^3^ Griffith University Brisbane Queensland Australia; ^4^ The University of Sydney School of Medicine Sydney New South Wales Australia

**Keywords:** antenatal care, patient perspective, qualitative study, stillbirth, stillbirth prevention

## Abstract

**Objective:**

This study explores women's and healthcare professionals' experiences and attitudes towards stillbirth risk and prevention conversations following Safer Baby Bundle (SBB) implementation. The SBB aimed to normalise these conversations within the antenatal healthcare setting.

**Design:**

A qualitative interview study.

**Setting:**

Maternity services in two states in Australia.

**Population of Sample:**

Eighteen postnatal women and 22 healthcare professionals at services that participated in the SBB.

**Methods:**

Qualitative study using semi‐structured interviews analysed using a deductive approach to thematic analysis.

**Main Outcome Measures:**

Themes identified around experiences of and attitudes towards antenatal care and conversations related to stillbirth risk and prevention.

**Results:**

Three key themes related to conversations about stillbirth in antenatal care were identified. First, ‘the importance of information that reassures and empowers’ through respectful communication, emphasising the ‘why’ of preventive actions, prioritising conversations over written information and positive framing. Second, ‘normalising sensitive conversations’ by reducing silence and stigma around stillbirth, having a standard way of doing things and shifting perceptions about the difficulty of raising this topic during pregnancy. Third, ‘steps towards respectful and supportive woman‐centred care’ influence the efficacy and responsiveness of conversations through continuity of care and carer for trust‐building and an ethos of addressing what matters most to women.

**Conclusions:**

Integrating conversations about stillbirth into routine antenatal care is facilitated by the SBB. Key considerations for effective, woman‐centred conversations about stillbirth include multidisciplinary collaboration, targeted HCP training and co‐designed resources to promote open communication, reducing fragmentation of care and a strengths‐based approach to discussing risk.

**Trial Registration:**

Australian New Zealand Clinical Trials Registry database: ACTRN12619001777189 (16 December 2019)

**Abbreviations**
CEQ: Clinical Excellence Queensland
CoC: continuity of care
FGR: foetal growth restriction
HCPs: healthcare professionals
NSW: New South Wales
QLD: Queensland
SBB: Safer Baby Bundle
Stillbirth CRE: Centre of Research Excellence in Stillbirth
VIC: Victoria

## 1 Introduction

Stillbirth is undeniably one of the worst outcomes for women and their families. Its potentially long‐lasting effects extend beyond the immediate family, impacting healthcare professionals who provide care and the broader healthcare system as a whole [1]. Enhancing awareness and understanding among women and their families about strategies to reduce the risk of stillbirth is key to reducing its incidence. Without these essential conversations, progress in the prevention of these tragedies remains unattainable. Despite ongoing efforts to improve communication, discussions surrounding stillbirth prevention in routine antenatal care often remain challenging and frequently avoided [2]. This persistent silence underscores a significant gap in current antenatal care practices.

In Australia, quality improvement through the implementation of a bundle of care: the Safer Baby Bundle (SBB) [3, 4] is a core strategy for advancing best practices in stillbirth prevention. The five evidence‐based SBB elements (smoking cessation, foetal growth restriction FGR, decreased foetal movements (DFM), side sleeping and optimal timing of birth) and their implementation are described elsewhere [3, 5]. The primary goal of the SBB is to reduce late gestation (≥ 28 weeks) stillbirth rates, aiming to support healthcare professionals (HCPs) to engage in open and informed conversations with women about their individual risk profile concerning stillbirth and how their care can be personalised accordingly [4, 5].

Maternity HCPs play a vital role in breaking the silence around stillbirth as trusted sources of support and information. Bereaved parents, perinatal loss support organisations and researchers have emphasised the importance of ‘normalising’ conversations about stillbirth and its risks as a public health priority to address the stigma and taboo [6, 7, 8, 9, 10]. However, concerns persist that discussing stillbirth creates unnecessary anxiety for pregnant women [11]. Studies in the United Kingdom [8] and Ireland [9] have highlighted the complexity of raising awareness of stillbirth during pregnancy. Despite this, research exploring the attitudes of both HCPs and women towards integrating these conversations into routine antenatal care is limited.

The SBB initiative supported standardising stillbirth conversations through training for HCPs and co‐designed resources for women to promote open communication. Since implementation, the proportion of HCPs routinely discussing stillbirth risks during antenatal care doubled from 35% to 83% (*p* < 0.001) [2].

This qualitative study aimed to explore the experiences and attitudes of HCPs and women regarding the provision and reception of information and care about stillbirth and its risks. Additionally, we aimed to examine how SBB implementation into services has supported integrating these conversations into routine antenatal care.

## 2 Methods

This qualitative sub‐study is part of a national maternity quality improvement initiative to reduce late gestation stillbirth. Maternity services from three Australian states—Queensland (QLD), New South Wales (NSW) and Victoria (VIC)—were recruited for state‐led SBB implementation programmes. The initiative and its mixed‐methods evaluation are described in the study protocol [5] and outlined briefly here.

### 2.1 Participants and Recruitment

Between August and December 2022, women and HCPs from 16 services that participated in SBB implementation programmes in NSW and QLD participated in interviews. On‐site SBB clinical leads assisted with recruiting eligible HCPs (midwives and doctors providing antenatal care) and women (who had given birth within the last 6 months, excluding those whose most recent pregnancy ended in stillbirth). Women were recruited postnatally either during their stay in hospital following birth, upon discharge or at a postnatal appointment. Participants received an AUD $30 gift card.

Details of HCPs and women were shared with the SBB Study Team, who contacted them to schedule interviews. Participants were provided an information sheet stating involvement was voluntary and withdrawal from the study would not affect workplace relations (HCPs) or care received (women).

### 2.2 Design

Semi‐structured interview guides were developed by a multidisciplinary team of researchers, HCPs and psychologists, and informed by surveys conducted before SBB implementation [2, 12]. The HCPs' interviews explored: attitudes towards the information and care provided to women regarding stillbirth risk (including the five SBB elements), their practices in discussing stillbirth and contextual factors influencing these conversations. The women's interviews focused on: overall impressions of their antenatal care and the information received relating to keeping their baby healthy before birth, awareness, knowledge and understanding of the SBB elements, and their feelings about receiving information on stillbirth risk and prevention. Demographic data were collected to provide transparency and contextualise the sample. Interview guides are provided in Appendices S1 and S2.

### 2.3 Data Collection, Analysis and Management

One‐to‐one interviews were conducted by A.L., C.A. and A.P. online (Zoom or Microsoft Teams) or via telephone. Interviews lasted 20–40 min, were recorded with consent, transcribed verbatim, de‐identified and imported into NVivo 12 for analysis. Recruitment continued until theoretical saturation was considered to have been reached, with recurring themes and no new patterns contributing to the narrative. Saturation was assessed separately for women and HCPs at the level of themes and sub‐themes and agreed upon through team discussions when no new insights were identified. Critical reflection on the role of the researchers in the study process is provided in Appendix S3.

We used thematic analysis that combined deductive and inductive approaches to align with the SBB evaluation questions, while allowing flexibility for new themes to be identified [13]. Four team members (C.A., D.R.F., L.S. and A.P.) reviewed all the transcripts and developed codes to connect concepts from the interviews with the research objectives. Meaningful conceptual themes supported by the data were developed from these codes through consensus. Other members (F.B. and V.J.F.) reviewed a subset of transcripts and appraised themes iteratively as they were developed.

Study data were securely stored on password‐protected drives on the Mater Research Institute‐University of Queensland network, with access restricted to the research team.

## 3 Results

### 3.1 Sample Characteristics

Forty participants (18 women and 22 HCPs) from 16 maternity services (QLD *n* = 6, NSW *n* = 10) were interviewed between 1 August and 31 December 2022. Summary characteristics of participants with study identifiers are listed in Tables S1 and S2.

Most women interviewed were born in Australia (68%, *n* = 12), had a previous pregnancy (61%, *n* = 11) and received either a public hospital (56%, *n* = 10) or midwifery group practice caseload (33%, *n* = 6) model of care. Of the 11 women who had a pregnancy before, nine had previous live births, two had previously experienced a stillbirth, four had experienced early pregnancy loss and five had not experienced any perinatal loss.

Most HCPs interviewed were female (95%, *n* = 21) and either midwives (64%, *n* = 14) or doctors (23%, *n* = 5).

### 3.2 Findings

Three major themes, each with sub‐themes, were identified (Table 1).

**TABLE 1:** Overview of identified themes and sub‐themes.

Themes	Sub‐themes
Importance of information that reassures and empowers	Connecting to the ‘why’ supports action.
	Conversations are important. We need to get better at having them.
Normalising sensitive conversations about stillbirth within antenatal care	Reduces the silence and stigma around stillbirth.
	Having a standard way of doing things made it easier for people to accept it as normal.
	Perception shifting from one of discomfort to importance of sensitive and open conversations.
Steps towards respectful and supportive woman‐centred care	Continuity of care and carer is important for relationship and trust‐building.
	Addressing what matters most to women.

#### 3.2.1 Theme 1: Importance of Information That Reassures and Empowers

A key theme identified was the provision and receiving of information about stillbirth, and its risks can be reassuring and empowering. Both women and HCPs highlighted the importance of connecting to the ‘why’ as central to supporting women to seek help when concerned during pregnancy. Additionally, prioritising open conversations over written information and using strengths‐based language in communications were viewed as more effective in providing reassurance. Illustrative quotes are presented in Table 2.

**TABLE 2:** Illustrative quotes for Theme 1.

Theme 1: Importance of information that reassures and empowers	
**Connecting to the ‘why’ supports action**	
1	‘I feel like, because we're giving this information all the time, they feel empowered to make that choice to ring up and go, yes, I am concerned. Like, it's not my baby's usual pattern’. (HCP 17, QLD, Registered Nurse/Midwife)
2	‘Yes. It did resonate with me, because I was a smoker, up until a couple of weeks before I found out I was pregnant. So, yes, I was very tuned in to that. It had just made my decision even more… I would never go back to it’. (Woman 12, QLD)
3	‘It's funny because we had discussions about that early on. I was sleeping on my side from 20 weeks onwards. When it was explained to me it was more rather something that can cause stillbirth, it was more about the reason why you sleep on your side’. (Woman 8, NSW)
4	‘It was all outlined and presented to us as this is what we need to do, and this is why we need to do it. This is the research behind it. Here are the tools that you may need, for example, with the elements about quitting smoking, it was here this might make life easier for you, to help with that’. (HCP 18, QLD, Midwife)
5	‘The e‐learning package helped me to introduce those conversations’. (HCP 19, QLD, Midwife)
**Conversations are important. We need to get better at having them**	
6	‘I think it was, kind of, just given to me as a bundle and they were like, any questions? Or like, are you a smoker? No, I'm not. Are, are you a drinker? No. Yeah, you know, about sleeping. Like, just, kind of, that very… Yeah, like, it was touched on but not in‐depth or anything like that’. (Woman 2, NSW)
7	‘Sometimes when I'm too busy I do just give the pamphlet to the woman. By giving them the pamphlet I can tick my box and say that I have talked about Safer Baby Bundle. But would a woman go home and read about it? I don't know’. (HCP 14, QLD, Obstetric Registrar)
8	‘We can hand out all the pamphlets under the sun, but sometimes they just get shoved in their antenatal booklet and don't get taken out’. (HCP 17, QLD, Registered Nurse/Midwife)
9	‘The conversation you have with women is much more in depth’. (HCP 8, QLD, Midwife)
10	‘Having conversations of not fear. It's more conversations about knowing, so that they can be more aware. And I think we've got a lot better at doing that. And I think that staff are a lot more comfortable’. (HCP 22, QLD, Clinical Midwifery Consultant)
11	‘… when they're telling the information like there are 2% chances of having that, or there are 10% chances of having that, they need to highlight the good information as well. They need to say, there are 90% chances to be good, you know, it matters. Because the statements can remain in our mind, so when they are saying there are 10% chances, the statement going to remain in our mind…So it will relieve. I think that's the way. Reassurance, reassurance’. (Woman 9, QLD)
12	‘You know, I mean, we've already talked about the risks of stillbirth, here's one of, you know, one of the other things that you can do to try and minimise your risks of that’. (HCP 7, NSW, Midwife)
13	‘I think first up. I think just to be informed straight up… You could then, sort of, do it gently and in stages’. (Woman 1, NSW)
14	‘Cause it's my first pregnancy, too much information. I couldn't deal with things in the starting. So, it was, kind of, stressful’. (Woman 4, NSW)

##### 3.2.1.1 Connecting to the ‘Why’ Supports Action

HCPs noted that women who understood the ‘why’ felt more confident asking questions and seeking help. As one midwife explained, when women understood the importance of being aware of their baby's regular movement pattern, they were better equipped with the knowledge of the actions to take when worried about foetal movements (Table 2, Quote 1).

Women reported that the information was presented in a clear and understandable way, helping them connect to the ‘why’ and how they could act. This was most evident for smoking cessation and side sleeping. A woman who recently quit smoking described how understanding the ‘why’ empowered and motivated her to remain smoke free (Table 2, Quote 2). Regarding going‐to‐sleep position, women noted that, although they initially switched to side sleeping during pregnancy for comfort, after discussing the rationale with their HCP, they viewed it more positively as a controllable and beneficial action for their pregnancy (Table 2, Quote 3). Similarly, HCPs felt the SBB resources and training helped them understand the evidence behind each element, giving them the confidence to prioritise information and facilitate open conversations (Table 2, Quotes 4 and 5).

##### 3.2.1.2 Conversations Are Important. We Need to Get Better at Having Them

Both women and HCPs agreed that brochures should never replace conversations with trusted HCPs. Some women felt the SBB brochures were given to them in place of meaningful conversations, leaving them unsure of their individual stillbirth risk (Table 2, Quote 6). HCPs shared a similar view, recognising that while brochures were useful tools for providing information, effective conversations are more important for promoting actions (Table 2, Quotes 7–9).

Both women and HCPs highlighted the importance of how conversations are framed. An experienced midwife emphasised the significance of the wording used to ensure information is conveyed to women respectfully, without causing fear (Table 2, Quote 10). While agreeing that receiving information about stillbirth was vital, several women shared that this did not ease their anxiety, particularly when information was presented in a way that focused on negative outcomes. As one woman (who previously experienced a stillbirth) explained, conversations were more reassuring when focused on the chance of positive outcomes rather than the risk of negative ones (Table 2, Quote 11).

Both HCPs and women stressed the importance of tailoring stillbirth discussions to each woman's individual needs and timing. Many felt conversations about stillbirth should begin early in the antenatal care continuum and then be revisited frequently (Table 2, Quotes 12 and 13). However, some concerns about information overload were raised, especially by first‐time mothers. One mother recalled feeling stressed at the beginning of her first pregnancy and felt the volume of information provided in early appointments compounded these feelings (Table 2, Quote 14).

#### 3.2.2 Theme 2: Normalising Sensitive Conversations About Stillbirth Within Antenatal Care

Participants indicated that when information and conversations about stillbirth are thought of as part of everyday antenatal care, they become normalised, shifting perceptions about the difficulty of raising this topic during pregnancy. Illustrative quotes are presented in Table 3.

**TABLE 3:** Illustrative quotes for Theme 2.

Theme 2: Normalising sensitive conversations about stillbirth within antenatal care	
**Reduces the silence and stigma around stillbirth**	
15	‘I think a lot more midwives are very comfortable now, talking about stillbirth risk with women. I don't know how often anybody ever used the word stillbirth before the Safer Baby Bundle actually came out’. (HCP 6, NSW, Midwife)
16	‘I think, just with the experience and the education, I think being able to talk to women about stillbirth and it not be such a hard task. It's just a part of normal education now. You know, before, it's obviously such a taboo subject, in general, and in society. But I think if we're talking about it at every visit, it just makes it more… just heaps more awareness and, hopefully, reduce the risks of it happening to them’. (HCP 17, QLD, Registered Nurse/Midwife)
17	‘I think that until you start making it a regular topic for everybody, then you're not going to get that spreading of information and acceptance that it's a topic that's safe to talk about and it's not a taboo topic. Until you make it a normal part of conversation. A normal part of the pregnancy journey’. (Woman 8, NSW)
18	‘You see miscarriage more often than like stillbirth, and you hear that more often like even from like the professionals around you. So, I think they could do more in terms of like telling people, this is a problem, this is something’. (Woman 7, NSW)
**Having a standard way of doing things made it easier for people to accept it as normal**	
19	‘Doing it in a multidisciplinary way. So, we knew, right from the get‐go, the doctors were talking about it, we were talking about it. There was lots of open communication across the team’. (HCP 19, QLD, Midwife)
20	‘It's made a huge difference. And I think really standardising our protocols, so the wording of them, and actually bringing in the bundle information to the work that we do every day so that it becomes normal jargon, doesn't become something unusual. And so, it's transitioned from, you know, something new that we have to do to be part of our day to day working, safety’. (HCP 1, NSW, Midwife)
21	‘Every time I go to the appointment…after my second trimester or something… they were really pressing me to know… That movements are really important’. (Woman 4, NSW)
22	‘Midwives like always say that at every midwife appointment. They just said, always, if you've got any concerns just ring…’ (Woman 6, NSW)
23	‘I feel it's pretty straightforward to use, because they're… A lot of them are relatively easy to implement into care. I mean, like, for the smoking one, was one I already discussed with patients anyway’. (HCP 11, QLD, Obstetric Registrar)
24	‘I don't really feel that the private women accessing private obstetrics are getting the same amount of information. They're definitely not getting the same language… If they ring up about decreased fetal movements, the obstetrician will usually just chuck a scanner on them in the room. Like, they won't do the bloods’. (HCP 1, NSW, Midwife)
25	‘Women are seeing their GPs six or seven times, and the GPs are not measuring their fundal height, they're not talking about the growth, or they're not talking about timing of the birth’. (HCP 10, QLD, Midwife)
**Perception shifting from one of discomfort to importance of sensitive and open conversations**	
26	‘Like, it's that accountability as a midwife to make sure that you have given that woman some really quick, but very, important information. Like, the talk doesn't take long. And often, I find by the time you say that women, they're, like, oh yeah, I know’. (HCP 1, NSW, Midwife)
27	‘So, being able to identify women who were at risk and then bring up those things and those terms and bring it up in a sensitive way, like, my practice has definitely changed. To be honest I probably wasn't really mentioning it before’. (HCP 19, QLD, Midwife)
28	‘Definitely it's the patient's right to know the information and it is the duty of the health provider to tell that information’. (Woman 9, QLD)

##### 3.2.2.1 Reduces the Silence and Stigma Around Stillbirth

Embedding conversations about stillbirth risk and prevention into routine care reduces the silence and stigma that often surrounds this topic. Some HCPs reflected that challenging the taboo nature of stillbirth had positively influenced their practice by improving their confidence in initiating open conversations without fear of increasing anxiety. This ensures they provide consistent information about risk reduction to all women. Two midwives specifically attributed the SBB to reducing the silence around stillbirth within their practice (Table 3, Quotes 15 and 16).

Women shared that hearing about stillbirth as part of their regular antenatal care took away the taboo nature of the topic and made them feel safe to talk about it with their HCP (Table 3, Quote 17). One woman (with a previous early pregnancy loss) compared this to her experience of HCPs more frequently speaking to miscarriage, feeling more could still be done to reduce the silence around stillbirth (Table 3, Quote 18).

##### 3.2.2.2 Having a Standard Way of Doing Things Made It Easier for People to Accept It as Normal

In services that implemented the SBB, stillbirth discussions have become largely accepted as ‘business as usual’ by HCPs. Both midwives and obstetricians said that having a standardised approach (facilitated by SBB) accelerated this acceptance (Table 3, Quote 19). They spoke about how the SBB supported practice change through standardising protocols, procedures and audits within multidisciplinary teams and health services. This instilled a high level of confidence that the information they are providing women will be consistent with their colleagues (Table 3, Quote 20). Two first‐time mothers highlighted the importance to them of receiving consistent information about foetal movements, regardless of which HCP they spoke with (Table 3, Quotes 21 and 22).

Several HCPs indicated they were already routinely performing many of the SBB recommendations, making adopting the SBB into their everyday practice easier. For example, smoking cessation was regularly discussed as an important action for a safer pregnancy (Table 3, Quote 23). Barriers to practice change were described, with HCPs highlighting a lack of awareness and uptake of the SBB within the private maternity system (private obstetricians and GP shared care). Some observed that women in the private system do not have access to the same standardised approach. Midwives from NSW and QLD indicated a lack of standardised processes and care for DFM and FGR among women receiving private care (Table 3, Quotes 24 and 25).

##### 3.2.2.3 Perception Shifting From One of Discomfort to Importance of Sensitive and Open Conversations

A shared understanding of the why and how of stillbirth risk communication was evident among women and HCPs, indicating a positive shift away from discomfort and towards recognising the importance of sensitive, open conversations about stillbirth. Both women and HCPs believed it was the duty of the provider to facilitate these open discussions (Table 3, Quotes 26–28).

#### 3.2.3 Theme 3: Steps Towards Respectful and Supportive Woman‐Centred Care

The SBB initiative was reported as well‐received and valued by most HCPs interviewed. Several raised contextual factors they felt influenced the efficacy and responsiveness of conversations about stillbirth risk. Illustrative quotes are presented in Table 4.

**TABLE 4:** Illustrative quotes for Theme 3.

Theme 3: Steps towards respectful and supportive woman‐centred care	
**Continuity of care and carer is important for relationship and trust‐building**	
29	‘I'm quite an anxious person. So, with the MGP group, seeing the same midwife, helped me. Just her presence of being there’. (Woman 15, QLD)
30	‘It was just so much easier knowing that I could toss out all my information, and she would just basically liaise on my behalf on…With a lot of things’. (Woman 14, QLD)
31	‘It really felt like we got personalised care. I felt that I was listened to. And that was really important because that first time around, you know, I was very shy, I didn't know what to expect. And she just provided us with so much information that was appropriate at each stage’ and ‘We had a very connected relationship with our midwife, so she knew us’. (Woman 8, NSW)
32	‘Every time I'm see someone, it's, like, always an introduction. You have to go through all the basics again. Yeah, or just develop that rapport’. In relation to discussing stillbirth with a known midwife ‘You'd be more open; there's already a relationship. You know? You'll be more honest of what you're feeling’. (Woman 17, QLD)
**Addressing what matters most to women**	
33	‘I guess it just comes down to, like, the communication side, and, just making the mums feel a little bit more, like, you're not just a number, I guess. You know, be a little bit more personalised, in that sense. And I know it's hard, and especially at this time, as well, but just giving that. But, also, giving that compassion when you're discussing those subjects and, the information as well’. (Woman 1, NSW)
34	‘I think because it was public health and, you know, you get someone different every time, and they're like so time constrained. They're just like. They literally run through all their questions, get their answers, and they're like, off you go. Like see you in two weeks’. (Woman 7, NSW)
35	‘At the end of the day, it was, whatever's going to be safest for the baby, and myself, obviously. But yes, whatever needed to happen so the baby would come out okay, then, that's what we needed to do’. (Woman 12, QLD)
36	‘It's such a short appointment time and there's, you've got so much to get to in Safer Baby from our hospital service. We're supposed to talk about almost all five elements every single appointment, and it's difficult in a just a standard 20‐minute appointment in the mainstream model’. (HCP 15, QLD, Midwife)
37	‘The information sometimes was an overload, and you just had to process, as much as possible’. (Woman 13, QLD)

##### 3.2.3.1 Continuity of Care and Carer Is Important for Relationship and Trust‐Building

Relationships and trust between HCPs and women when discussing stillbirth and its risks were highly valued and thought to be essential for improving conversations. Women who received midwifery continuity of care (CoC) reported feeling less anxious discussing sensitive topics, suggesting a potential influence of the model of care on comfort and openness during interactions (Table 4, Quotes 29–31). One woman reflected that her appointments always felt rushed. She believed that if she ‘had her own midwife’, they might have been more accommodating due to an existing relationship, which would also make discussing sensitive topics such as stillbirth more meaningful (Table 4, Quote 32).

##### 3.2.3.2 Addressing What Matters Most to Women

When asked what matters most to them, women were consistent in emphasising the necessity of feeling heard and understood by their care provider. They expressed a strong desire for a safe space where they could openly discuss their thoughts, feelings, and concerns without fear of judgement. One woman shared that she wanted her care to be personalised, rather than feeling like just another person in the waiting room (Table 4, Quote 33). The challenge of this was recognised, acknowledging antenatal appointments are under time constraints, with one woman feeling her care was less meaningful when her midwife just ran through a checklist of questions at her appointments (Table 4, Quote 34).

One first‐time mother echoed the feelings of many, highlighting that the most important aspect of her care was the reassurance that her baby was safe and healthy and that every measure would be taken to ensure this (Table 4, Quote 35). Components both women and HCPs indicated may hinder respectful and supportive woman‐centred care included HCPs acting as ‘gatekeepers’ who restricted access to knowledge and information, limited time in appointments with competing priorities and overloading women with too much information at once (Table 4, Quotes 36 and 37).

## 4 Discussion

### 4.1 Main Findings

This qualitative study offers unique insights into women's and HCPs' attitudes towards routinely discussing stillbirth and its risk reduction during pregnancy, highlighting how implementing the SBB can support normalising sensitive conversations.

We found a shared understanding and acceptance among both women and HCPs that conversations around stillbirth during pregnancy are a necessary and important part of antenatal care. SBB implementation into standard practice drives conversations about stillbirth risk and prevention. Using the SBB initiative and resources to address stillbirth openly and consistently across multidisciplinary teams, HCPs feel more confident initiating conversations without fear of causing undue anxiety for women. This approach ensures all women receive consistent information, which is crucial for reducing stillbirth risks. Women, in turn, appreciated the accessibility and clarity of information tailored to their individual needs, even though this occasionally caused some apprehension.

### 4.2 Strengths and Limitations

A strength of the study is that interviews with women about the antenatal care they received were conducted soon after birth, allowing them to reflect on their full pregnancy care experience. However, women could not clearly recall every aspect of their antenatal care, and recollections could have been influenced by subsequent experiences with labour, birth or postpartum care.

Another study's strength is the exploration of perspectives from both women and HCPs, with themes drawn from their combined experiences, providing a balanced understanding of attitudes shaping conversations and care. This was strengthened by interviewing women who received different models of care and service locations, and HCPs with varying years of experience and primary work areas. Whilst contrasts were observed between women who experienced different models of care and parity, other demographic factors appeared to have limited influence on the themes identified. Interview findings corroborated our survey results from the same population [2], enhancing the validity of our conclusions. Additionally, women interviewed had diverse pregnancy experiences, suggesting the transferability of findings to other maternity care users and contexts. However, most women interviewed were Australian‐born English‐speaking, and the perspectives of those from different cultural backgrounds may not be represented. While no clear culturally specific differences emerged in our current findings, we acknowledge the limited cultural diversity in our sample population. Understanding and meeting the needs of women from diverse cultural backgrounds enables the provision of best‐practice care and information sharing around stillbirth awareness and prevention [14]. In Australia, the risk of stillbirth is higher for some communities, including Aboriginal and Torres Strait Islander women and some women from migrant and refugee backgrounds [15]. Cultural adaptations of the SBB have now been released for Indigenous families (*Stronger Bubba Born*) and migrant and refugee families (*Growing a Healthy Baby*) and evaluation is underway.

One limitation of this study is that recruitment was managed exclusively by on‐site clinical teams, preventing documentation of the number approached or differences between participants and decliners. This process may have introduced selection bias. Those with stronger attitudes towards care experiences or the SBB initiative may have been more likely to be invited or agree to participate. As a result, findings may disproportionately reflect the views of those more inclined to be informed or engaged, potentially biasing results and limiting their broader applicability. Interviewers' backgrounds in psychology and perinatal health may have influenced data collection and interpretation, and some team members were involved in the development and rollout of the SBB, which may have shaped perspectives; however, regular team discussions and diverse disciplinary input supported reflexivity and helped minimise bias.

### 4.3 Interpretation

This study addresses the stigma and silence surrounding stillbirth in maternity care and the broader community. In line with Pollock et al. [16], who emphasised the importance of breaking the silence around stillbirth, our findings suggest that when HCPs engage more openly with the topic, it can enhance their confidence in initiating sensitive conversations and ensure the consistent delivery of risk‐reduction information to all women. Several HCPs reflected that this shift and discussing stillbirth more transparently has positively influenced their practice, allowing them to support women without fear of increasing anxiety. These qualitative insights build on our earlier survey findings that, before SBB implementation, HCPs often avoided discussing stillbirth with low‐risk pregnant women, fearing unwarranted distress [2]. Our findings show that women value being informed about stillbirth risk and prevention, even if it involves potential anxiety. This aligns with an Irish study exploring first‐time mothers' attitudes towards the provision of information about stillbirth, which suggested that silence around stillbirth risks does little to meet the needs of women and their families and limits understanding about the link to changes in behaviour for reducing stillbirth risk [9]. This can lead to women feeling uninformed and seeking potentially less reliable sources (such as websites and apps) that may provide conflicting or less evidence‐based information [17, 18]. Furthermore, our study underscores the necessity for HCPs to provide this information consistently and sensitively, ensuring clear and supportive communication.

The theme of connecting to the ‘why’ demonstrates the importance of providing information that enables informed decision‐making, fostering a sense of control and reassurance. By explaining the evidence and reasons behind recommendations and actions, women are more inclined to take proactive steps when concerned during their pregnancy. Similarly, HCPs' knowledge and practice gaps are a commonly reported barrier to guideline implementation [19]. This study indicates that when HCPs are informed about the research supporting their recommendations, they gain confidence, which in turn supports more effective communication. For example, concerning foetal movements, the literature suggests HCPs' attitudes and knowledge vary widely, leading to inconsistent practice [20, 21]. Additionally, women have reported being dismissed or made to feel like a nuisance when contacting their HCPs with concerns about movements [11]. Benefiting from targeted support to standardise practice, our findings demonstrate the importance of appropriate co‐designed resources and training for enhancing communication to promote behavioural shifts. Some women also described instances where information was delivered in a checklist‐like manner or through brochures without discussion, suggesting that even well‐intentioned communication can fall short without meaningful engagement. This highlights the need for future enhancements to the SBB to include training that supports more personalised, interactive conversations.

Aligning with the SBBs' approach to reduce variations in care by implementing elements with strong clinical support (expected routine care) [22], HCPs interviewed indicated many recommendations were already performed before implementation. Additionally, establishing a standard approach facilitated easier adoption and acceptance by multidisciplinary teams. Our findings provide a deeper understanding of how the SBB has changed stillbirth risk conversations between HCPs and women. Prior to the SBB, many HCPs avoided these discussions, perceiving them as uncomfortable or stressful [2, 23]. Reframing these conversations as integral to antenatal care, based on a shared understanding of the importance of sensitive communication, has shifted this mindset. We identified that respectful, direct and strengths‐focused conversations about stillbirth help women receive the information in a reassuring and empowering way. This shift in perception has improved overall experiences of care, making it more inclusive of vital risk discussions. These qualitative insights extend previous survey findings that reported an increase in the frequency of stillbirth conversations following SBB implementation [2]. This study offers novel contributions by exploring the underlying factors that made these conversations more acceptable and effective, specifically the role of trust, CoC and positively framed, woman‐centred communication. These findings go beyond documenting increased frequency to explain *why* these conversations were better received and how they contributed to more meaningful and supportive care experiences.

Women in this study emphasised the importance of feeling respected and supported by HCPs when discussing sensitive topics like stillbirth. They valued supportive communication and the ability to express their concerns without feeling dismissed. CoC and carer play a critical role in building trust and strong relationships between HCPs and women [24]. Thus, a core principle underlying the SBB was enhancing access to CoC for all pregnant women, particularly those identified as high risk for stillbirth [25]. Similar to a study on midwifery CoC for women at higher risk of preterm birth [26], our findings show that building trusting relationships can reduce anxiety levels when discussing stillbirth. Additionally, we highlight how the midwife‐woman relationship facilitates effective, responsive conversations about stillbirth, fostering personalised care, trust and empowerment. This conclusion is supported by a recent systematic review of qualitative studies on what women value within the midwifery CoC model [24].

Reducing fragmentation of maternity care has been identified as an important clinical practice improvement area to reduce stillbirths [27]. Enhancing access to CoC in the public health system, including within and between teams, can improve the efficacy and responsiveness of conversations about stillbirth risk. For HCPs, feeling supported through multidisciplinary teamwork to provide consistent, respectful care was essential, as was the shared view that it is the HCP's duty to facilitate open discussions about stillbirth and its risk factors as part of best practice. Additionally, to improve integration across the maternity care system, future implementation of the SBB should consider strategies such as targeted outreach to better engage private sector providers and ensure consistent messaging and resource access across all models of care.

## 5 Conclusion

Stillbirth is a highly sensitive topic and one that has been surrounded by silence and stigma. Open conversations may help mitigate modifiable risk factors associated with stillbirth, potentially reducing the likelihood of such tragic outcomes. The Australian SBB has taken steps to support HCPs to incorporate these discussions and standardised information into routine antenatal care. Our findings suggest that this structured approach is well received by women and HCPs. However, consistent with the principles of women‐centred care, careful attention and a personalised approach are critical. To effectively integrate woman‐centred conversations about stillbirth into routine antenatal care, we recommend: multidisciplinary collaboration for cohesive information sharing; targeted training for healthcare professionals; co‐designed resources to promote women's understanding and open communication; relationship building through CoC; and a strengths‐based approach to discussing stillbirth risk and prevention.

## Author Contributions

C.A. led development of the study protocol and interview guides, the conduct of the study, conducted some interviews, analysis and interpretation of data and prepared the manuscript. A.P. coordinated and conducted interviews, assisted in the preparation of the manuscript and approved the final version. F.B. assisted in the design of the interview guides, analysis and interpretation of data, reviewed the manuscript and approved the final version. A.L. conducted most of the interviews, reviewed the manuscript and approved the final version. D.R.F. and L.S. assisted in the analysis and interpretation of data, reviewed the manuscript and approved the final version. D.A.E. and A.G. assisted in study design, reviewed the manuscript and approved the final version. V.J.F. conceived the study, oversaw the development of the study protocol and tools, the analysis plan and interpretation of the results, assisted in the preparation of the manuscript and approved the final version.

## Ethics Statement

Human research ethics approval was obtained from the Royal Brisbane & Women's Hospital Human Research Ethics Committee (HREC) in June 2019 (approval number: HREC/2019/QRBW/47709). The processes used by this HREC to review multi‐centre research proposals have been certified by the National Health and Medical Research Council. Verbal consent to the study was obtained after informing participants of the purpose of the interviews and their privacy rights. The study protocol outlining the planned evaluation of the SBB across maternity services in New South Wales (NSW), Queensland (QLD) and Victoria (VIC) has been published [5].

## Consent

The authors have nothing to report.

## Conflicts of Interest

The authors declare no conflicts of interest.

## Supporting information


**Appendix S1:** Safer Baby Bundle study, post‐implementation: clinician interviews.

**Appendix S2:** Safer Baby Bundle study, post‐implementation: women (and partners if available) interview.

**Appendix S3:** Reflexivity statement.

**Table S1:** Characteristics of interview participants (women and HCP).

**Table S2:** Individual participant characteristics.

## Data Availability

The data that support the findings of this study are available on request from the corresponding author. The data are not publicly available due to privacy or ethical restrictions.
