# Supplementary material for: Conversations About Stillbirth Risk in Routine Antenatal Care: A Qualitative Study Post‐Implementation of the Safer Baby Bundle
Source: BJOG. 2025 Aug 13;132(12):1856–65. doi: 10.1111/1471-0528.18330 (PMC12501658; doi:10.1111/1471-0528.18330)
Supplement: Supplementary file 1 — Appendix S1: Safer Baby Bundle study, post‐implementation: clinician interviews. [file BJO-132-1856-s003.rtf]

Safer Baby Bundle Study, Post Implementation: Clinician interviews
Welcome
Thank you for agreeing to be interviewed. We appreciate your time and willingness to be involved.
Introductions
My name is (interviewer) and I am a researcher from the Stillbirth Centre for Research Excellence. We are conducting interviews with clinicians who are involved with the antenatal care of women from maternity services that participated in implementing the Safer Baby Bundle (SBB). 
The Purpose of the Interviews
We are interested in your experience with implementing the five SBB elements of care in your practice. The overall aim of this study is to gain an in-depth account of individual clinician's perspectives of the SBB initiative and resources– how it went and how it could be improved. Your involvement will help researchers evaluate antenatal care before and after the SBB and will help to improve care for pregnant women in Australia.
The interview should take about 20-30 minutes.  

In line with the Privacy Act, your personal details are confidential. Information that could identify you will not be linked to your responses.  The information you share with us will only be used for research purposes. Your participation in this study is completely voluntary, if, at any time you want to stop the interview, we can do that without any reason. I will be audio recording this interview as I want to capture everything you have to say. This will be used to produce a de-identified transcript to assist the researchers with their analysis. We think there are probably a lot of different experiences and opinions – and that is why we are doing these interviews. So, we'd really like your input and would like you to share your honest and open thoughts with us.

Do you have any questions before we start? 
Do you consent to proceed with the interview? Yes/No


Note- Interviewer will have access to pre-interview background information collected including: State, maternity service they currently work at and discipline (e.g. midwife, Obstetrician).

Firstly, a few questions about you and the maternity service you work in.
Service Characteristics- Can you tell me a little about the maternity service at “------name of Hospital----“?
·	Size- Number of births annually? 
·	Remoteness- Major city/Inner Regional/ outer regional/remote/very remote. 
·	Service Capability- Level
Interviewee Characteristics- Can you tell me about yourself, your primary work area and your level of experience
·	Discipline (Obstetrics/Midwifery, GP…)- known from pre-interview Q's
·	Primary work area (Midwifery group practice, pregnancy assessment unit, antenatal inpatient…)
·	Years of service
Now, a few questions regarding the information and care women receive regarding the five SBB elements of care. 
1.	Awareness- Before being asked to take part in this interview, had you heard about the Safer Baby Bundle Initiative or seen any information about the SBB? 
·	How were you first made aware of the SBB (Prompt- Attend a launch day/study day, was it incorporated into in-service education, peer to peer coms, newsletter, CRE)
·	How familiar are you with the five elements of the SBB? Can you please explain what they mean to you in your clinical practice.
·	What education and/or training have you received across the SBB and the five elements?
2.	What is your overall impression of the SBB initiative?
3.	Has the SBB led you to change any aspects of your own practice in relation to the information and care you provide women regarding stillbirth risk and how women can reduce their risk? 
·	Can you tell me about this? 
·	How has it changed the conversations you have with women about risk of stillbirth? 
·	What aspects were the most difficult to change and what were the easiest?
·	Did you have enough time/support from your service
4.	Follow up Question if need more SBB element specific = More specifically, has the SBB led you to change any aspects of your own practice in relation to the five elements of care- for example- referral or smokers to Quitline, FGR risk screening and surveillance, management of women who present with concerns about fetal movements etc. 
Now, let's talk about your maternity service and experiences with implementing the SBB
5.	What facilitative/helpful factors do you believe have assisted in implementation of the SBB program at your service, and why?
·	Prompts and follow on question if needed: To what extent are these factors generic (could be applied to other programmes) or context specific (only relevant to your program)? E.g., Enough resources, manpower, IT.)
6.	What barriers or blocks to implementation do you believe exist, and why? 
·	Prompts and follow on question if needed: (Things that have hindered the programme in some way?). Is there anything delaying or preventing implementation?

7.	Do you think the SBB recommendations of care are now embedded into your everyday practice and will be sustainable? Will some elements be more challenging than others for you to sustain?
o	Prompt and follow on questions if needed: To what extent are any factors generic (could be applied to other programmes/initiative) or context specific (only relevant to SBB work)?

8.	What impact do you think the COVID 19 pandemic has had on implementing the SBB at your service? 
·	Can you also tell us about some of the ways your delivery of antenatal care has changed due to COVID and are these changes likely to remain in place?
·	Prompts and follow on question if needed: To what extent was any impact on SBB implementation efforts generic (could be applied to other programs) or context specific (i.e. certain SBB elements of care). SFH measurement- did women start measuring at home
9.	Were you satisfied with the SBB resources, is there anything we can improve?
·	Resources for clinicians- eLearning, education sessions, care pathways
·	Resources for women- brochures, videos, social media
10.	Are there any other care practice/s that you feel could be improved to reduce stillbirths and neonatal deaths?
11.	Any final comments about the Safer Baby Bundle initiative or the information and care you provide to women and their families to keep babies healthy before birth.
Thank you for sharing that information with us. We at the CRE offer our sincere thanks to you and your colleagues for all your hard work and for helping to reduce stillbirth. Thank you for your time today.

Appendix 1: For reference
Background information- Pre-interview at scheduling phone call or via email
Before your interview we would like to confirm a little bit of background information. This information is to help us understand the data when we analyse this information later on, however we will not analyse or report any individual information that may identify you. If you would prefer not to answer any of the following demographic questions, please just let me know by saying 'prefer not to say' for that question.
1.	In which state or territory is your maternity service located?
2.	In which maternity service (study site) do you currently work in?
3.	What is your professional discipline? (obstetrics, midwifery, GP, other?)
4.     What gender do you currently identify with? (Male, female, other, prefer not to say)
