# Supplementary material for: Conversations About Stillbirth Risk in Routine Antenatal Care: A Qualitative Study Post‐Implementation of the Safer Baby Bundle
Source: BJOG. 2025 Aug 13;132(12):1856–65. doi: 10.1111/1471-0528.18330 (PMC12501658; doi:10.1111/1471-0528.18330)
Supplement: Supplementary file 2 — Appendix S2: Safer Baby Bundle study, post‐implementation: women (and partners if available) interview. [file BJO-132-1856-s005.rtf]

Safer Baby Bundle Study, Post Implementation: Women (and partners if available) interview
Welcome
Thank you for agreeing to be interviewed. We appreciate your time and willingness to be involved.
Introductions
My name is (interviewer) and I am a researcher from the Stillbirth Centre for Research Excellence. We are conducting interviews with women about their experiences with antenatal care in maternity services that participated in the Safer Baby initiative. 
The Purpose of the Interviews
The reason we are doing these interviews is to gather the experiences of women around different aspects of antenatal care and the information received about keeping your baby healthy before birth. We will be discussing issues related to the health of your baby before birth. 
Fortunately, Australia is one of the safest countries in the world to have a baby, but tragically, around six babies are born still every day. The Safer Baby team are working hard to ensure fewer parents experience this unimaginable loss. This study is focussing on improving care through better information and education for women during pregnancy and for their health care providers about risk factors for stillbirth and steps to take to reduce the risk. Your involvement will help researchers evaluate antenatal care before and after the SBB is introduced and will help to improve care for pregnant women in Australia.
The interview should take about 30 minutes.  

In line with the Privacy Act, your personal details are confidential. Information that could identify you will not be linked to your responses.  The information you share with us will only be used for research purposes. Your participation in this study is voluntary, if at any time you want to stop the interview, we can do that without any reason. I will be audio recording this, as I want to capture everything you have to say. We do not identify anyone by name in our report. You will remain anonymous. We think there are probably a lot of different experiences and opinions – and that is why we are doing these interviews. So, we'd really like your input and would like you to share your honest and open thoughts with us.

If at any time during this interview you feel worried about the discussion, you are encouraged to talk to the researchers at the time and to seek advice from your care providers. You can also contact the Pregnancy, Birth and Baby Helpline by calling 1800 882 436 or visiting www.health.gov.au/pregnancyhelpline.

Do you have any questions before we start? 
Do you consent to proceed with the interview? Y/N


Note- Interviewer will have access to pre-interview background information collected including: State, hospital received antenatal care and gave birth, age, ethnicity, country of birth, previous pregnancies (outcomes)- see background information form. Prompt- confirm anything that is unclear.

Firstly, a question about the model of antenatal care you had: 

Antenatal care- What was your main model of antenatal care? (Prompt- Public hospital care/ Private obstetrician / Private midwifery / General Practitioner shared care/ Midwifery group/team practice/ Midwifery caseload /Other)

Now, I would like to ask you some questions related to the information and care you received before your baby's birth (throughout your pregnancy). 
1.	Can tell us your overall impression of the care and information you received from you doctor or midwife about keeping your baby healthy before birth?
2.	What mattered most to you during your antenatal appointments?   

For this study, we are particularly interested in aspects of your care associated with the Safer Baby initiative. 
3.	Before being asked to take part in this study, had you heard about the Safer Baby Initiative or seen any information about the Safer Baby? (If yes, can you tell me about this?, if No- that's OK, your experiences with care are very important to us, it just helps us to know what information you might have seen). 

4.	At any stage during your pregnancy did you discuss or were you provided with any information about risk factors for having a stillbirth? How did you feel about this? (Prompt- if they answer no- is there any type of information or discussion would you have liked to have had about reducing the risk of having a stillbirth?)

5.	Do you think providing information to pregnant women about the factors which are known to increase the risk of stillbirth is important? If so, when is an appropriate time for women to receive this information?

6.	General SBB: During your pregnancy did you receive any information or advice about the five topics included in the Safer Baby? 
a.	If yes- what was your impression of this information, was it helpful, who gave it to you? How could they be improved?
b.	If no- or don't know what these are- The five topic areas of the SB include information and advice around: smoking in pregnancy, monitoring your baby's growth, how to interpret a change in your baby's movements, safe sleeping position during the third trimester and making a plan for the timing of your baby's birth?

Now, I would like to ask you some questions specific to the five topics included in the Safer Baby program  
7.	Smoking: Did you smoke during your pregnancy (even if only occasionally) or did anyone regularly smoke inside your home or car? Can you tell us about any information and/or advice you received from your midwife/doctor about smoking (/or passive smoking) during pregnancy. (prompt- did they mention stillbirth as a risk, were you offered a referral, did you uptake the referral, how did you feel about this discussion).

8.	Sleep: Did you try to avoid going-to-sleep in any particular position in late pregnancy? 
·	If yes- can you tell me more about this? (prompt- did your midwife or doctor discuss with you what a safe going-to-sleep position is in late pregnancy? Was this information helpful or unhelpful? Brochure? Improvements?
·	If no- were you given a brochure or did your midwife or doctor discuss with you what a safe going-to-sleep position is in late pregnancy (after 28 weeks' gestation)? Would you have liked to have been given this information, if so when?)

9.	DFM: During your pregnancy, did you experience any concerns about your baby's movements? If so how confident did you feel to discuss this with your health service provider?( Follow up for Yes- did you contact your HCP, did you visit your HCP, at what gestation was this, what care did you receive, how did you feel about this?) (Follow up for No- if you had felt concerns about movements how confident would you have felt to discuss this with your health service provider (prompts- straight away or delay until next appoint, felt like bothering them etc).
10.	FGR: During your pregnancy, did you experience any concerns about your baby's growth? Did your midwife/doctor measure the size of your abdomen using a tape measure? Yes/No
a.	Did you midwife/doctor talk with you about next steps if there were signs that your babies growth has slowed? How did you feel about this? Were you given enough information? Where you sent for any extra tests or ultrasound scans?

11.	TOB 1: During the last trimester of your pregnancy, did you have a conversation with your doctor or midwife about planning the best time for your baby to be born? (ie. scheduled date and time for birth rather than waiting for labour to start on its own). If so, how did you feel about this? (Prompts- confident, supported, included, provided with enough information, risk and benefits discussed in a way you could understand etc.). 
(Extra wording- Giving birth close to your due date is generally best for your baby's development. However, sometimes babies need to be born at an earlier time and this is called planned birth. The main reason women have a planned birth is to reduce the chance of problems for them or their baby, including stillbirth.)

12.	TOB 2: Deciding whether you should have a planned birth or wait for your baby to be born is not always easy. It is important to know the benefits and risks of both options for you and your baby. Do you have any suggestions for how these conversations could be improved?

13.	Information Source: Throughout pregnancy what was your main source for information about keeping your baby healthy before birth? Apps, advice from your doctor, family, social media?

14.	OPTIONAL: One of the recommendations of the Safer Baby to support women and their partners to stop smoking during pregnancy is to offer all women a breath test at their booking visit to measure the level of carbon monoxide. This is regardless of whether they identify as smokers or not.
a.	****ONLY ask at health service where known CO monitors in use at time women would have received antenatal care. Were you offered a breath test to measure the level of carbon monoxide? When were you offered a breath test and how did you feel about this? 
b.	***if no CO in use due to COVID or not implemented then…. Your maternity service was not able to do this part of the study due to the impact of COVID. If you had been asked to take a breath test do you have any thoughts on how you might have felt about this at the time?
15.	COVID- If not already mentioned- do you have any reflections on how COVID- 19 may have impacted on your care or your experience with antenatal care.
16.	Any final comments about the Safer Baby initiative, your care or the information you received about keeping your baby healthy before birth?

Thank you for sharing that information with us. We at the CRE offer our warmest congratulations to you and your family on the birth of your baby and thank you again for participating in our research. We will be sending you a $30 e-gift voucher via email, this can take a few days to process, so if you haven't received anything with in a week, please contact us.

Appendix A. For reference only
Background information- Pre-interview at scheduling phone call or via email
Before your interview we would like to gather a little bit of background information. This information is to help us understand the data when we analyse this information later on, however we will not analyse or report any individual information that may identify you. If you would prefer not to answer any of the following demographic questions, please just let me know by saying 'prefer not to say' for that question.
(prompt- some of these details may be known e.g. State and maternity service, so will just need to confirm).
1.	In which state or territory, did you receive most of your antenatal care?
2.	Through which hospital/maternity service did you receive most of your antenatal care? 
3.	At which hospital did you give birth?
4.	With what ethnicity do you identify with?
5.	What is your country of birth?
6.	What was your age (years) at time of giving birth (this pregnancy)?
7.	Prior to this most recent pregnancy have you been pregnant before? Y/N
a.	If yes, can you briefly tell me about this/these?
