# Supplementary material for: Conversations About Stillbirth Risk in Routine Antenatal Care: A Qualitative Study Post‐Implementation of the Safer Baby Bundle
Source: BJOG. 2025 Aug 13;132(12):1856–65. doi: 10.1111/1471-0528.18330 (PMC12501658; doi:10.1111/1471-0528.18330)
Supplement: Supplementary file 3 — Appendix S3: Reflexivity statement. [file BJO-132-1856-s004.docx]

## **Reflexivity Statement**

The research team that conducted this study includes experts in implementation science, psychology, midwifery, obstetrics, neonatology, social sciences, bereavement care and health services research. All interviewers received training in qualitative methods and interview techniques. Interviews were conducted by; AL, who is a female registered psychologist with a background in perinatal mental health; CA, who is a female postdoctoral researcher with a background in perinatal health services research; and, AP, who is a female research assistant with a Masters of Global Public Health and currently studying a Bachelor of Midwifery. The following members of the research team assisted with data analysis and interpretation of transcripts, but did not conduct interviews; DF, who is a male PhD candidate with a background in nursing; and, LS, who is a female research assistant with a background in social science. Senior clinician researchers from both jurisdictions included in this study assisted with high level interpretation of findings; DE, who is a male Obstetrician specialising in high-risk pregnancies from QLD; and, AG who is a female staff specialist Neonatologist from NSW.

FB and VF who oversaw study-related tasks to monitor for quality and rigour are highly experienced researchers who have been involved in multiple qualitative studies. Researchers were not involved in the clinical care of the women nor the workplace of HCPs who participated.
