# Supplementary material for: Conversations About Stillbirth Risk in Routine Antenatal Care: A Qualitative Study Post‐Implementation of the Safer Baby Bundle
Source: BJOG. 2025 Aug 13;132(12):1856–65. doi: 10.1111/1471-0528.18330 (PMC12501658; doi:10.1111/1471-0528.18330)
Supplement: Supplementary file 4 — Table S1: Characteristics of interview participants (women and HCP). [file BJO-132-1856-s002.docx]

**Table S1** Characteristics of Interview Participants (Women and HCP)

| **Women (n=18)** | | | |
| --- | --- | --- | --- |
|  | | n | % |
| **State** | NSW | 8 | 44% |
|  | QLD | 10 | 56% |
| **Ethnicity** | Aboriginal or Torres Strait Islander | 1 | 5% |
|  | African | 1 | 5% |
|  | Asian | 4 | 22% |
|  | Caucasian | 12 | 68% |
| **Country of birth** | Australia | 12 | 68% |
|  | France | 1 | 5% |
|  | India | 3 | 17% |
|  | Philippines | 1 | 5% |
|  | South Sudan | 1 | 5% |
| **Prior pregnancy** | Yes | 11 | 61% |
|  | No | 7 | 39% |
| **Model of care** | Public Hospital | 10 | 56% |
|  | Midwifery group practice caseload | 6 | 33% |
|  | Shared Care (with GP) | 1 | 6% |
|  | Private midwifery care | 1 | 6% |
| **Maternity Healthcare Professionals (n = 22)** | | | |
|  | | n | % |
| **State** | NSW | 7 | 32% |
|  | QLD | 15 | 68% |
| **Location of service** | Major city | 9 | 41% |
|  | Regional centre | 3 | 14% |
|  | Regional | 10 | 45% |
| **Discipline/Role** | Midwife | 14 | 64% |
|  | RN/Midwife | 3 | 13% |
|  | MD (Registrar and Obstetrics-Gynaecologist) | 5 | 23% |
| **Gender** | Female | 21 | 95% |
|  | Male | 1 | 5% |
