# Supplementary material for: Conversations About Stillbirth Risk in Routine Antenatal Care: A Qualitative Study Post‐Implementation of the Safer Baby Bundle
Source: BJOG. 2025 Aug 13;132(12):1856–65. doi: 10.1111/1471-0528.18330 (PMC12501658; doi:10.1111/1471-0528.18330)
Supplement: Supplementary file 5 — Table S2: Individual participant characteristics. [file BJO-132-1856-s001.docx]

Table S2. **Individual Participant Characteristics**

| **Women** | **Identifier** | **State** | **Age** | **Country of birth** | **Prior pregnancy** | **Model of Care** | **Maternity Service location** |
| --- | --- | --- | --- | --- | --- | --- | --- |
|  | W1 | NSW | 25-29 | Australia | Yes | Midwifery group practice | Major city |
|  | W2 | NSW | 25-29 | Australia | Yes | GP shared care | Major city |
|  | W3 | NSW | 30-34 | India | No | Public hospital | Regional centre |
|  | W4 | NSW | 25-29 | India | No | Public hospital | Regional centre |
|  | W5 | NSW | Under 20 | Australia | Yes | Midwifery group practice | Regional centre |
|  | W6 | NSW | 20-24 | Australia | No | Public hospital | Regional centre |
|  | W7 | NSW | 20-24 | Australia | Yes | Public hospital | Regional centre |
|  | W8 | NSW | 35-39 | Australia | Yes | Private midwifery | Major city |
|  | W9 | QLD | 30-34 | India | Yes | Public hospital | Major city |
|  | W10 | QLD | 30-34 | Australia | No | Midwifery group practice | Regional centre |
|  | W11 | QLD | 30-34 | Australia | No | Public hospital | Regional centre |
|  | W12 | QLD | 25-29 | Australia | No | Public hospital | Major city |
|  | W13 | QLD | 30-34 | Australia | Yes | Public hospital | Regional centre |
|  | W14 | QLD | 25-29 | South Sudan | Yes | Public hospital | Regional centre |
|  | W15 | QLD | 20-24 | Australia | No | Midwifery group practice | Regional centre |
|  | W16 | QLD | 40 and over | Australia | Yes | Midwifery group practice | Major city |
|  | W17 | QLD | 30-34 | Philippines | Yes | Public hospital | Major city |
|  | W18 | QLD | * | France | Yes | Midwifery group practice | Regional centre |
| **HCPS** | **Identifier** | **State** | **Discipline** | **Years of Service** | **Primary work area** | | **Maternity Service location** |
|  | HCP 1 | NSW | Midwife | >10 years | Birth Suite | | Major city |
|  | HCP 2 | NSW | Midwife | >10 years | Clinical Midwifery Consultant | | Major city |
|  | HCP 3 | NSW | Midwife | >10 years | Antenatal Clinic | | Regional centre |
|  | HCP 4 | NSW | Registered Nurse/Midwife | >10 years | Clinical Midwifery Educator | | Regional centre |
|  | HCP 5 | NSW | Midwife | >10 years | Birth Suite | | Regional centre |
|  | HCP 6 | NSW | Midwife | * | Clinical Midwifery Educator | | Regional centre |
|  | HCP 7 | NSW | Midwife | 5-10 years’ | Clinical Midwifery Specialist | | Regional centre |
|  | HCP 8 | QLD | Midwife | 5-10 years’ | Maternal Fetal Medicine | | Major city |
|  | HCP 9 | QLD | Registered Nurse/Midwife | >10 years | Antenatal Clinic | | Regional centre |
|  | HCP 10 | QLD | Midwife | >10 years | Antenatal Clinic | | Major city |
|  | HCP 11 | QLD | Registrar | 5-10 years’ | Maternal Fetal Medicine | | Major city |
|  | HCP 12 | QLD | Registrar | <5 years’ | Obstetrics | | Regional centre |
|  | HCP 13 | QLD | Obstetrician Gynecologist | 5-10 years’ | Obstetrics | | Major city |
|  | HCP 14 | QLD | Registrar | <5 years’ | Obstetrics | | Regional centre |
|  | HCP 15 | QLD | Midwife | >10 years | Antenatal Clinic/Community Midwifery Services | | Regional centre |
|  | HCP 16 | QLD | Midwife | >10 years | Antenatal Clinic | | Major city |
|  | HCP 17 | QLD | Registered Nurse/Midwife | >10 years | Antenatal Clinic | | Regional centre |
|  | HCP 18 | QLD | Midwife | * | Maternity Inpatient Unit | | Regional centre |
|  | HCP 19 | QLD | Midwife | 5-10 years’ | Clinical Midwifery Consultant | | Regional centre |
|  | HCP 20 | QLD | Midwife | 5-10 years’ | Maternity Unit | | Major city |
|  | HCP 21 | QLD | Registrar | <5 years’ | Obstetrics | | Major city |
|  | HCP 22 | QLD | Midwife | >10 years | Clinical Midwifery Consultant | | Regional centre |

*Missing data
